# Magnitude and associated factors of contrast induced nephropathy among patients undergoing coronary angiography and interventions at a cardiac referral hospital in Tanzania - a cross-sectional study

**DOI:** 10.11604/pamj.2021.38.311.24536

**Published:** 2021-03-29

**Authors:** Pilly Chillo, Ng´wigulu Malaja, Peter Kisenge

**Affiliations:** 1 Department of Internal Medicine, Muhimbili University of Health and Allied Sciences, Dar es Salaam, Tanzania,; 2 Department of Cardiology, Jakaya Kikwete Cardiac Institute, Dar es Salaam, Tanzania

**Keywords:** Contrast induced nephropathy, acute kidney injury, Tanzania, sub-Saharan Africa

## Abstract

**Introduction:**

contrast media are increasingly used in diagnostic and interventional procedures but are also known causes of acute kidney injury - a condition known as contrast induced nephropathy (CIN). We aimed to determine the magnitude and associated factors of CIN among patients undergoing coronary angiography and percutaneous coronary intervention at a cardiac referral hospital in Tanzania.

**Methods:**

all adult patients undergoing elective coronary angiography and percutaneous coronary intervention at Jakaya Kikwete Cardiac Institute were consecutively enrolled between August 2017 and January 2018, if they fulfilled the inclusion criteria. Pre-procedure, 24- and 72-hours' post procedure serum creatinine was measured. CIN was defined as increase of ≥25% or absolute increase of ≥44μmol/L of serum creatinine within 72 hours following exposure to contrast media. Data analysis were done using SPSS Version 20. P-value of <0.05 was considered statistically significant.

**Results:**

in total, 210 (94.6%) out of 222 patients seen during the study period fulfilled the inclusion criteria and were enrolled. Their mean (SD) age was 61.3 (10.9) years and 64.3% were men. Hypertension, diabetes, smoking and alcohol consumption was present in 86.7%, 37.7%, 12.4% and 37.6% respectively. The incidence of CIN was 19% within 72 hours post procedure. On multivariate logistic regression analysis, independent factors for developing CIN were history of heart failure (aOR=7.34), central obesity (aOR=3.12), triple vessel disease (aOR=10.14) and post procedure stay of ≥3 days (aOR=4.1), all p<0.05.

**Conclusion:**

the incidence of CIN found in this population is high (19%) and is associated with heart failure, obesity, multi-vessel disease and longer post-procedure hospital stay.

## Introduction

The growing use of imaging and interventional procedures that involve the administration of intravascular contrast media in sub-Saharan Africa [1] will significantly increase the number of patients at risk to develop contrast induced nephropathy (CIN). CIN is defined as the impairment of renal function, measured as either a 25% increase in serum creatinine from baseline or 44 μmol/L increase in absolute serum creatinine within 72 hours of intravenous contrast administration [2]. In large observational studies conducted in North America, CIN has been established as the third most common cause of hospital-acquired acute kidney injury, after impaired renal perfusion and use of nephrotoxic medications [3,4]. The frequency of occurrence of CIN differs depending on the procedure performed as well as the studied population. Coronary angiography and percutaneous coronary interventions have been associated with highest rates of CIN, reported to occur in 11 - 16% among unselected cardiac patients [5] and as high as 50% among sub-groups of cardiac patients with high-risk [6].

Despite an increase in the use of contrast media, CIN has been largely understudied in sub-Saharan Africa, with only a few studies reported from South Africa, Kenya and Nigeria [7-9] while in Tanzania no documented literature exist regarding CIN. The previous studies from the region reported an incidence of CIN ranging from 9.9% to 35.9% [7-9] with most of the patients being exposed to contrast media following radiological investigation of contrast enhanced computed tomography. In the study by Banda *et al*. from South Africa, presence of CIN was associated with longer hospital stay and mortality at 3 months, indicating adverse effects of CIN among native sub-Saharan Africans [7]. With regards to risk factors associated with the development of CIN, several studies have documented clinical factors including pre-existent chronic kidney disease, diabetes mellitus, advanced age, anemia, heart failure, metabolic syndrome, hypoalbuminemia and dehydration, as well as factors related to the procedure performed and the contrast medium used [10]. However, reports have been inconsistent in risk factors for CIN [11] and more need to be done to generate local data.

In Tanzania, as it is for many sub-Saharan African countries coronary artery diseases has been increasing [12] and in 2016 alone the Jakaya Kikwete Cardiac Institute (JKCI) performed coronary angiography and/or interventions in 620 patients and the numbers are increasing. It is therefore likely that CIN will pose a significant challenge both for cardiologists and nephrologists. This study was carried out to document the magnitude and associated factors of CIN among patients undergoing percutaneous coronary angiography and interventions at the Institute, in order to obtain local baseline data to inform preventive measures.

## Methods

**Study area, population and period:** the study was carried out at JKCI, which is the national referral hospital for cardiac conditions. The hospital has a bed capacity of 104 and an average of 5 patients undergoing coronary angiography or intervention daily. All adult patients (≥18 years) who underwent elective coronary angiography and/or percutaneous coronary intervention at JKCI during the study period were invited to participate. Patients on dialysis and those who did not give consent to participate were excluded. The study was conducted between August 2017 and January 2018.

**Study design and sample size:** this was an observational, hospital-based cross section study. Patients were consecutively enrolled in the study until a pre-defined sample size of 210 patients was reached, which was sufficient to estimate the prevalence of CIN at 5% error margin and power of 80%, considering the previous known prevalence of CIN in South Africa [7].

### Data collection procedure

**Questionnaire and data collection forms:** a structured questionnaire was used to collect patients´ information, which included socio-demographic characteristics (age, sex, marital status etc), cardiovascular disease history, drug history, pre-existing renal disease and history of previous exposure to contrast medium. Patients´ hospital case notes were used to determine other co-morbidities, as well as pre-procedure laboratory results of the patient. Information from patients´ notes was recorded into pre-coded data collection forms.

**Physical examination, anthropometric and blood pressure measurements:** patients´ height and weight was examined and recorded. Height was measured by use of a stadiometer with patients wearing no shoes and recorded to the nearest centimeter. Weight was measured by using pneumatic weighing machine (Mormet®, China) and recorded to the nearest 0.5 kg. Height and weight were used to calculate body mass index (BMI). Obesity was defined as BMI ≥30 kg/m^2^. Waist circumference was measured by use of a tape measure and recorded to the nearest centimeter. Central obesity was defined as waist circumference >88 cm for females and >102 cm for males [13]. Blood pressure was taken using an automated BP machine. Measurements were taken pre-procedure, intra-, at 24hr- and 72hr-post procedure. Hypertension was defined as pre-procedure BP of ≥140/90 mmHg or known hypertensive on medications.

**Laboratory investigations and definition of CIN:** serum creatinine was measured within 24 hours before the procedure and at 24- and 72-hours post procedure. CIN was defined as an absolute increase of serum creatinine of 44 μmol/l or a 25% increase from pre-procedural serum creatinine level [2]. Serum creatinine levels were used to calculate the estimated glomerular filtration rate (eGFR) using the modification of diet in renal disease (MDRD) Study equation [14] and patients were categorized into different stages of chronic kidney disease (CKD) as per guidelines [15]. Patient´s hemoglobin level, serum cholesterol and fasting blood glucose were done within 24 hours before the procedure. All tests were done at the Muhimbili National Hospital Central Pathology Laboratory which is the reference laboratory for Tanzania.

**Data processing and analysis:** data entry and analysis were done using Statistical Package for Social Sciences (SPSS) Version 20.0 software. Data is expressed as mean (SD) for continuous variables and as number (%) for categorical variables. Groups of patients were compared using Chi-square test or Fisher´s exact test for categorical variables and unpaired students´ t-test for continuous variables. Multivariate logistic regression analysis was performed to determine independent factors associated with CIN among study patients. A two tailed p-value of less than 0.05 was considered to indicate a statistically significant difference.

**Ethical issues:** ethical clearance was obtained from the Research and Publications Committee of Muhimbili University of Health and Allied Sciences. All patients had to sign an informed consent form before any data were collected. Patients were free to choose to be part of the study, refusal of which did not interfere with their right to get service at the hospital.

## Results

**Socio-demographic and clinical characteristics of the study population:** during the study period, 222 patients fulfilled the inclusion criteria and were enrolled but only 210 had a second blood sample for creatinine analysis post procedure. Among the 12 patients without second creatinine results; 2 died before the second creatinine sample was collected, 5 had lost creatinine results and 5 did not come for the second creatinine measurements.

Patients´ characteristics are summarized in Table 1. Of the 210 patients analyzed, 135 (64.3%) were men. The mean (SD) age of the total population was 61.3 (10.9) years (range 33 - 86 years) and most of the patients were in the age group 41 - 65 years. Hypertension was the most prevalent cardiovascular risk factor, being present in 86.7% of the total population. Previous history of heart failure and of renal failure was present in 3.8% and 8.6% respectively, while history of previous coronary angiography procedure was present in 22.6% of the patients. Majority of the study patients had good renal function pre-procedure. None of the study participants was using the known nephrotoxic drugs namely aminoglycosides, amphotericin B or cyclosporine.

**Table 1:** socio-demographic, clinical and laboratory findings

Characteristic	Frequency	%
**Gender**		
Men	135	64.3
Women	75	35.7
**Age groups (years)**		
18 - 40	8	3.8
41 - 65	128	61.0
>65	74	35.2
**Cardiovascular risk factors**		
Known hypertensive	182	86.7
Known diabetics	75	37.7
Taking alcohol	79	37.6
Cigarette smokers	26	12.4
**Previous cardiovascular disease**		
History of heart failure	8	3.8
History of renal failure	18	8.6
Previous angiography procedure	42	22.6
**Medications used**		
Diuretics	113	53.8
ARBs	105	50.0
CCBs	46	21.9
ACEIs	29	13.8
NSAIDs	4	1.9
Obese (BMI ≥30kg/m2)	82	39.0
Central obesity	119	56.7
**Hypertension stage**		
Normal/controlled BP	108	51.4
Stage 1	71	33.8
Stage 2	23	11.0
Stage 3	8	3.8
Anemia	60	28.6
High cholesterol	68	33.5
Elevated FBG	66	31.6
**Pre-procedure CKD stage**		
eGFR >60	164	78.1
Stage 1	38	18.1
Stage 2	7	3.3
Stage 3	1	0.5

ARB: angiotensin converting enzyme inhibitor; CCB: calcium chanel blocker; ACEI: angiotensin converting enzyme inhibitor; NSAID: non-steroidal anti-inflammatory drug; FBG: fasting blood glucose; BMI: body mass index; BP: blood pressure

**Magnitude and associated factors of CIN:** Table 2 summarizes the trends of serum creatinine from baseline and within 72 hours post procedure. At baseline, the mean serum creatinine of the total population was 105.4 ± 48.3 μmol/l. It slightly increased at 24 hours post-procedure (110.4 ± 57.7 μmol/l) and came back to pre-procedural levels at 72 hours (104.2 ± 54.1 μmol/l) post procedure. At 24 hours post procedure 10 (5%) patients had an absolute serum creatinine increase of ≥44 μmol/l while 24 (12.1%) patients´ serum creatinine increased by ≥25% from baseline. It is noted that all 10 patients with a ≥44 μmol/l absolute increase of serum creatinine from baseline met the ≥25% serum creatinine increase criteria, making the incidence of CIN at 24 hours post procedure to be 12.1% (Table 2).

**Table 2:** trends of serum creatinine from baseline to 24- and 72-hours post procedure

	Baseline (N=210)	At 24 hours (N=199)*	At 72 hours (N=200)*
Mean ± SD creatinine (μmol/l)	105.4 ± 48.3	110.4 ± 57.7	104.2 ± 54.1
Mean ± SD eGFR (ml/min/1.73m2)	78.8 ± 26.4	77.2 ± 27.3	81.8 ± 27.7
Proportion with increased creatinine, n (%)	NA	105 (52.8)	98 (49.0)
Proportion with decreased creatinine, n (%)	NA	92 (46.2)	102 (51.0)
Proportion with un-changed creatinine, n (%)	NA	2 (1%)	0 (%)
Proportion with ≥44μmol/l, n (%)	NA	10 (5%)	13 (6.5%)
Proportion with 25% increase, n (%)	NA	24 (12.1%)	25 (12.5%)
Proportion with CIN, n (%)	NA	24 (12.1%)	26 (13.0%)

eGFR: estimated glomerular filtration rate; CIN: contrast induced nephropathy; *only 199 and 200 patients had creatinine measurements at 24 hours and 72 hours respectively; NA: not applicable

**Figure 1:**
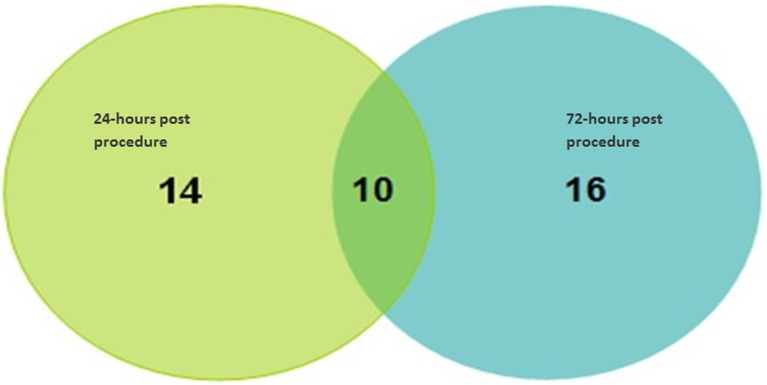
venn diagram showing the distribution of 40 patients with CIN

The overall incidence of CIN, defined as absolute serum creatinine increase of ≥44 μmol/l from baseline and/or ≥25% increase of serum creatinine from baseline within 72 hours post angiography procedure was present in 40 (19.05%) patients. Of the 40 patients who developed CIN, 10 had CIN both at 24 hours and at 72 hours (Figure 1). Furthermore, 14 of the 24 patients with CIN at 24 hours had normal creatinine at 72 hours, while 16 out of the 26 patients with CIN at 72 hours had normal creatinine at 24 hours. None of the patients diagnosed to have CIN needed dialysis.

Table 3 compares the socio-demographic, clinical and peri-procedural characteristics between patients who developed CIN and those who did not. Patients with CIN did not differ from those without CIN in most of the socio-demographic and clinical characteristics. As seen in Table 3, age, gender, history of hypertension, mean blood pressure levels, proportion of diabetes, smoking or alcohol consumption did not significantly differ between patients with CIN and in those without CIN, all p>0.05. Furthermore, mean eGFR as well as proportion with anemia did not differ between the two groups. However, patients with CIN were more likely to have a history of heart failure, previous myocardial infarction and were more likely to have had received fluid therapy pre-procedural, with longer mean duration of the procedure as well as longer hospital stay post procedure (Table 3).

**Table 3:** socio-demographic, clinical and peri-procedural characteristics of patients with and without CIN

Characteristic	CIN (n=40)	No CIN (n=170)	p-value
**Socio-demography**			
Females, n (%)	14 (35.0)	61 (35.9)	0.917
Mean ± SD age (years)	63.1 ± 11.6	60.9 ±10.8	0.244
Age ≥65 years, n (%)	18 (45.0)	63 (37.1)	0.226
**Clinical**			
Hypertensive, n (%)	35 (87.5)	147 (86.5)	0.863
≥Grade 2 hypertension, n (%)	5 (12.5)	26 (15.3)	0.654
Overweight and obese, n (%)	33 (82.5)	133 (78.2)	0.551
Central obese, n (%)	27 (67.5)	92 (54.1)	0.124
Diabetics, n (%)	15 (37.5)	60 (35.3)	0.793
Mean ±SD fasting blood sugar (mmol/l)	7.2 ± 4.0	6.5 ± 2.9	0.221
Anemic, n (%)	14 (35.0)	46 (27.1)	0.314
Elevated FBG, n (%)	13 (32.5)	53 (31.4)	0.889
Mean ±SD eGFR (mL/min/1.73 m2)	84.5 ± 32.3	77.4 ± 24.8	0.128
CKD stages, n (%)			
eGFR > 60	33 (82.5)	131 (77.1)	0.868
Stage 3 CKD	6 (15.0)	32 (18.8)	
Stage 4 CKD	1 (2.5)	6 (3.5)	
Stage 5 CKD	0 (0.0)	1 (0.6)	
Taking alcohol, n (%)	15 (37.5)	64 (37.6)	0.986
Smoking, n (%)	6 (15)	20 (11.8)	0.576
History of heart failure, n (%)	4 (10)	4 (2.4)	0.023
History of renal failure, n (%)	4 (10)	14 (8.2)	0.120
Taking NSAIDs, n (%)	0 (0)	4 (2.4)	0.327
Taking ACEIs or ARBs, n (%)	21 (52.5)	113 (66.5)	0.098
Taking diuretics, n (%)	24 (60)	89 (52.4)	0.383
**Peri-procedure**			
Had myocardial infarction, n (%)	15 (37.5)	30 (17.6)	0.006
Received angiography + PCI, n (%)	8 (20.0)	29 (17.1)	0.660
Pre-procedural fluid given, n (%)	8 (20.0)	10 (5.9)	0.004
Procedure duration ≥20min, n (%)	14 (35.0)	35 (20.6)	0.053
Contrast volume ≥100ml, n (%)	10 (25.0)	20 (11.8)	0.031
3 vessel disease on CAG, n (%)	7 (17.5)	4 (2.4)	<0.001
≥3days post-procedure hospital stay, n (%)	18 (45.0)	26 (15.3)	<0.001

Results are mean ± SD unless stated otherwise; CIN: contrast induced nephropathy; NSAIDs: non-steroidal anti-inflammatory drugs; ACEIs: angiotensin converting enzyme inhibitors, ARBs: angiotensin receptor blockers; FBG: fasting blood glucose

Factors found to be associated with CIN (p<0.05) and those with weak associations (p≤0.25) in univariate analysis (Table 3) were entered in logistic regression model to determine independent factors associated with the development of CIN. These factors were age ≥65, central obesity, history of heart failure, history of renal failure, taking angiotensin converting enzyme inhibitors/angiotensin II receptor blockers (ACEI/ARB), previous myocardial infarction, having pre-procedure IV fluids, procedure time >20 minutes, volume of contrast >100 ml, having 3-vessel disease on angiogram, and having >3 days post procedure hospital stay. Gender (female vs male) was then forced in the model as an important confounder. In the final logistic regression analysis (after removing interacting variables), the independent factors associated with development of CIN were history of heart failure (aOR=7.34, CI 1.55 - 34.83), central obesity (aOR=3.12, 95% CI 1.22 - 7.97), triple vessel disease on angiogram (aOR=10.14, 95% CI 2.07 - 49.65) and longer (≥3 days) post-procedure hospital stay (aOR=4.09, 1.75 - 9.76) irrespective of age and gender, all p<0.05 (Table 4).

**Table 4:** independent factors associated with CIN among patients undergoing percutaneous coronary angiography and interventions at JKCI

Variable	cOR (95% CI)	p-value	aOR (95% CI)	p-value
Age ≥65 (years)	1.4 (0.69 - 2.78)	0.354	1.41 (0.63 - 3.13)	0.405
Female gender	0.96 (0.47 - 1.98)	0.917	0.88 (0.38 - 2.01)	0.756
History of heart failure	4.6 (1.10 - 19.31)	0.036	7.34 (1.55 - 34.83)	0.012
Central obesity	1.8 (0.85 - 3.64)	0.127	3.12 (1.22 - 7.97)	0.017
Given IV fluids pre-procedural	4.0 (1.47 - 10.92)	0.007	2.43 (0.72 - 8.25)	0.154
Contrast volume ≥100 mls	2.5 (1.06 - 5.87)	0.036	1.58 (0.55 - 4.47)	0.396
Three vessel disease on CAG	8.8 (2.44 - 31.79)	0.001	10.14 (2.07 - 49.65)	0.004
≥3 days hospital stay	4.5 (2.14 - 9.59)	<0.001	4.1 (1.75 - 9.76)	0.001

CIN: contrast induced nephropathy; CAG: coronary angiography

## Discussion

This study documents for the first time, that the incidence of CIN among un-selected patients undergoing coronary angiography and percutaneous coronary intervention at the National Cardiac Referral Hospital in Tanzania is 19%. Furthermore, the development of CIN in this population is independently associated with pre-existing history of heart failure, presence of central obesity, multi-vessel disease and longer post-procedure hospital stay. The magnitude of CIN found in this study is lower than that reported by the study done in Nigeria among patients undergoing radiocontrast procedures (35.9%) [9]. The incidence found in our study is however, higher than reports from South Africa and Kenya [7,8]. Differences in the patients´ characteristics and procedures performed could explain some of the variations between ours and previous studies from the region. Our findings however, falls not far from the incidence reported in a review of literature by Gami *et al*. which reported an incidence of CIN ranging between 11 - 16 % following coronary angiographic procedures in large studies in Europe and North America [5]. The finding that a pre-existing history of heart failure was independently associated with the development of CIN has been reported elsewhere in developed [16-18] as well as in developing countries [19,20]. Our study confirms this finding and adds to the sub-Saharan Africa literature that patients with heart failure are up to 7 times more at risk to develop CIN when compared to those without heart failure. The mechanism of CIN in heart failure has been described to be a result of poor pre-renal perfusion which results in a deleterious effect of renal vasoconstriction coupled with low preload that compromise the medullary oxygenation; and in the presence of contrast media this leads to increased nephrotoxicity and hence CIN [10].

Obesity, especially central obesity, is characterized by the presence of metabolic syndrome, itself a risk factor for renal impairment [21]. Previous studies have documented the association of obesity and/or metabolic syndrome and the development of CIN among patients undergoing any contrast administration [22] as well as those undergoing coronary angiography procedures [23,24]. However, others have dismissed this association as an independent risk factor [25]. The mediators of abnormal kidney function in obesity include physical compression of the kidneys by fat in and around the kidneys, activation of the renin angiotensin aldosterone system and increased sympathetic nervous system activation [26]. Of note, obesity was not included as a risk factor in the South African study [7] and in the Nigerian study obesity was not associated with CIN [9]. Our findings therefore need to be confirmed by other researchers in the region.

Patients with triple vessel disease are generally known to have accelerated or diffuse atherosclerosis which is linked to the development of CIN [18,27,28]. We found in this study that patients with triple vessel disease were 10-times more likely to get CIN when compared to those with lesser coronary artery disease. Apart from the deranged renal function, patients with triple vessel disease have impaired pumping ability of the heart which further impairs renal perfusion. Yet, another explanation is the fact that these patients require multistage angiography and/or interventions thus utilizing high volume of contrast media [29]. In fact, high volume of contrast medium was associated with CIN in univariate analysis in this study. Our finding therefore confirms similar observations from previous studies [16,30]. Although this study did not collect data on mortality, it is likely that patients with triple vessel disease and CIN will have worse long term outcomes similar to what was seen by Abe *et al*. in Japan, where triple vessel disease predicted CIN and was associated with increased longer term mortality after PCI [30].

We found patients who stayed longer in hospital (for three days or more) had at least 4 times higher likelihood to develop CIN irrespective of other variables. This observation agrees with the findings by previous researchers in Europe and North America [31,32]. However, the study from South Africa by Banda *et al*. which studied patients admitted due to any cause did not find this association, most likely due to the different patient population studied [7]. Longer hospital stay is an outcome of CIN and indicates the general poor condition of the patient. In the study by Marenzi *et al*. in Italy, development of CIN was strongly associated with longer hospital stay, complicated the clinical course and it was a predictor of increased mortality in that study population [32].

Our finding that patients who received fluids pre-procedure were more likely to develop CIN in univariate analysis deserves a special mention. This finding has been reported by others [7] and can be explained by preferential intravenous fluid administration in at risk patients as a preventive measure for CIN. Nevertheless, in tightly controlled studies as is the case for randomized control studies, hydration has proved to be a preventive measure to develop CIN [33,34]. In South Africa, the incidence of CIN was 15.8% and 26.3% in the pre-hydrated and non-hydrated patients, respectively [7].

In this study age, diabetes mellitus, pre-existing renal impairment as well as anemia were not associated with the development of CIN. This is in contrary to previous studies [7,9,16,18,35,36]. It is likely that our study population was not powered to detect these differences and only trends were observed in univariate analyses. These lack of associations have however, been as well observed in the few research on CIN in Africa. For example, age, diabetes and pre-existing renal failure were all not associated with CIN in the studies by Banda *et al*. in South Africa as well as in the study by Radwan *et al*. in Egypt [7,37]. Larger regional studies are therefore needed to look into the details of these observations. The strengths of this work include that this is the first study to report on the prevalence and risk factors of CIN in Tanzania. The prospective nature of the study gives a true picture of what happens to patients receiving contrast medium within 72 hours post cardiac angiographic procedures in our center. The study is limited since it is not possible to report on the long-term outcome of the patients with CIN as this was not part of the present study.

## Conclusion

The incidence of CIN among patients undergoing coronary angiogram or percutaneous coronary intervention at the Jakaya Kikwete Cardiac Institute is high (19%) and is independently associated with positive history of heart failure, central obesity, multi-vessel disease and longer post-procedure hospital stay.

### What is known about this topic

- Use of contrast media for investigations as well as interventional procedures is increasing worldwide and in sub-Saharan Africa;
- Patients receiving contrast media are at risk to develop contrast induced nephropathy, which carries a risk to long-term renal damage and mortality.

### What this study adds

- We document for the first time that CIN is present in 19% of patients undergoing coronary angiography and interventions at a cardiac referral hospital in Tanzania; this incidence is high but falls within the documented prevalence in sub-Saharan Africa of 9.9% to 35.9%, depending on the studied population;
- This study adds that, patients with pre-existing heart failure, obese and those with multiple coronary vessels disease carry higher risk to develop CIN.
